# Fatal Dog Maulings in Colorado: A Forensic Case Series

**DOI:** 10.7759/cureus.90756

**Published:** 2025-08-22

**Authors:** Rayan Zarei, Leon Kelly

**Affiliations:** 1 Department of Biomedical Sciences, Rocky Vista University College of Osteopathic Medicine, Parker, USA

**Keywords:** animal control, canine-induced trauma, comparative case series, elderly victims, fatal dog attacks, forensic pathology, multi-dog mauling, public health policy, scene investigation, wound morphology

## Abstract

Lethal dog attacks, though comparatively uncommon, are a serious forensic, public health, and medico-legal problem. Our case series explores three lethal canine attacks occurring in the first months of 2025 in the state of Colorado as a way of better understanding canine-mediated mortality. Autopsy and investigative reports were analyzed for three adult victims who were killed over six weeks as a result of dog attacks. Cases were identified through review of official coroner autopsy records, with inclusion limited to deaths certified as due to canine mauling; all other cases were excluded. Demographic data, scene context, and injury findings were extracted from the autopsy reports and investigative files. Comparative analysis was conducted to assess similarities and differences in injury distribution, environmental context, and contributing health factors. Case 1 involved a 76-year-old woman with dementia, fatally attacked by her own 14 dogs at home, suffering exsanguination from femoral artery trauma. Case 2 concerned a 68-year-old woman with multiple comorbidities, found with extensive defensive wounds and avulsion injuries in a suspected feral or uncontained canine attack. Case 3 described a 57-year-old man fatally mauled while walking his dog, with major scalp avulsion and bite injuries inflicted by two pit bulls. All deaths were certified as accidental. Common forensic findings included patterned puncture wounds, soft tissue gouging, and circumferential limb injuries. Defensive wounds were present in two of the three cases. Scene contexts ranged from domestic residences to public areas, with both owned and unrestrained dogs involved. Here, the volume of cases in a short period of time demonstrates the forensic significance of the determination of canine injury patterns, specifically among susceptible victims and street canine populations. This comparative analysis provides insight into wound morphology, victim response, and issues involving multi-canine attack scenes. These findings may have an impact on future guidelines of forensic investigation, animal control laws, and health education.

## Introduction

Deadly canine attacks are a rare and tragic intersection of forensic pathology, public health, and animal control. An estimated 30-50 deaths yearly are thought to occur as a result of canine aggression in the United States alone, with hundreds of others gaining disfiguring injuries or injuries altering the life of the relevant victim irreparably [1,2]. While the violence and unpredictability of such events draw considerable media attention, the forensic literature guiding systematic study of mortality due to dog mauling is scant, notably when comparative analysis of multiple cases occurs at the local jurisdiction level [3,4].

It is difficult to investigate a death related to a dog from a medicolegal perspective. Dog-provoked injuries are often similar to sharp or blunt force injuries, and scene evidence could be obscured by postmortem scavenging or lack of eye-witness accounts [5,6]. Cause and manner of death are ascertained by considering the pattern of injuries, the presence of defensive injuries, the breed of the dogs, the number of dogs, and the medical history of the deceased [7,8].

This report thus undertakes a comparative forensic analysis of three deadly canine attack incidents that were perpetrated during the earlier months of 2025, all of which occurred in the state of Colorado. The events range from domestic pet bites within the premises to multi-dog bites outside. Through autopsy records, scene analysis, and broader epidemiologic comparison, these incidents can contribute to the scant literature of deaths related to canines.

## Case presentation

Case 1

A 76-year-old female who had a history of dementia was discovered deceased inside her residence in Colorado City, CO, on February 3, 2025. Scene investigation uncovered that the deceased had been residing with roughly 14 canine pets of assorted breeds: Corgis, English Bulldogs, Cavalier King Charles Spaniels, and Boxers. According to her daughter, the decedent was left alone while errands were run earlier that afternoon. Upon return later in the day, she was found unresponsive and covered in blood, lying partially in the hallway and partially in the bedroom. Emergency responders noted extensive visible injuries and no signs of life.

**Figure 1:**
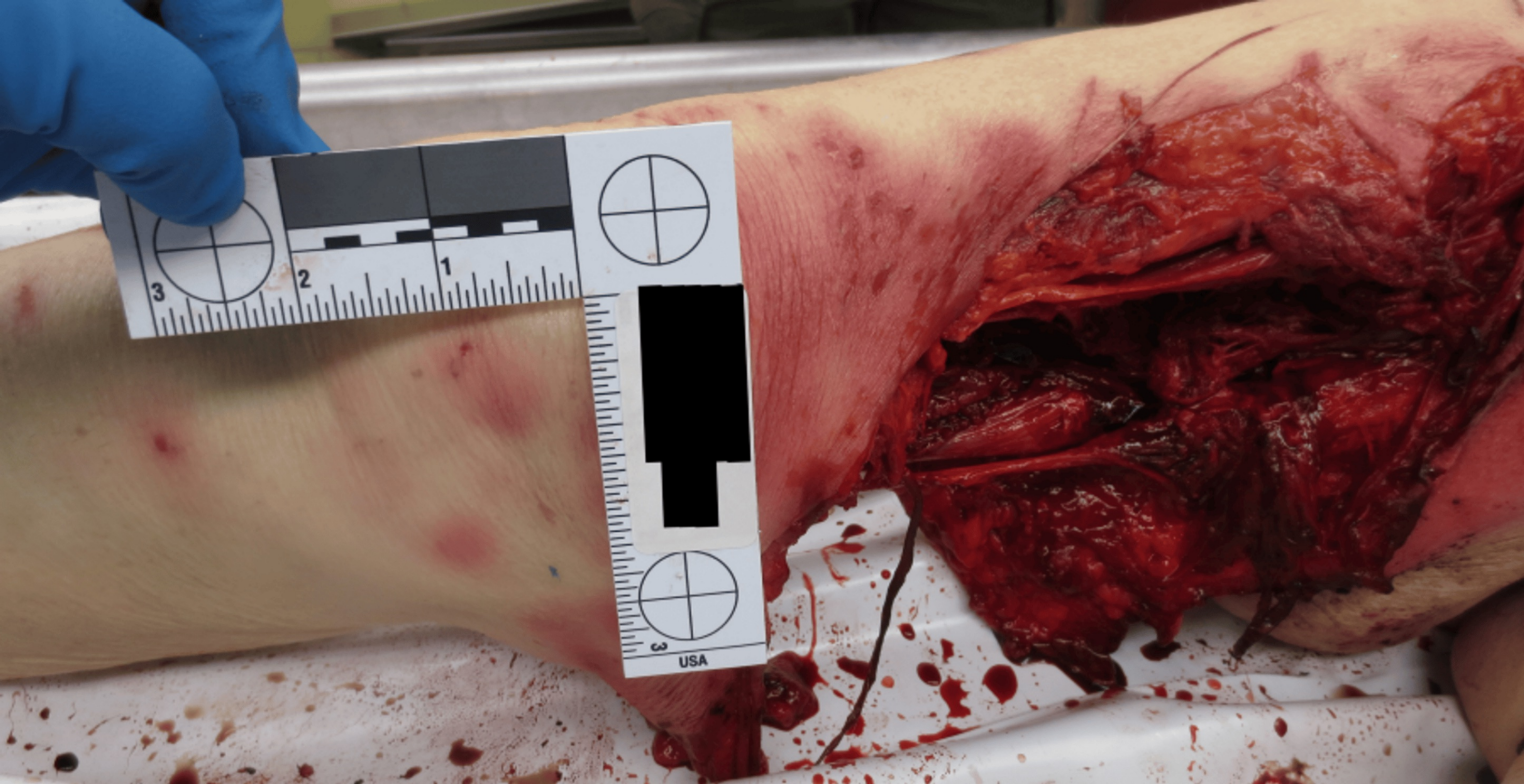
Avulsion injury to the right medial thigh with transection of the femoral artery and vein.

**Figure 2:**
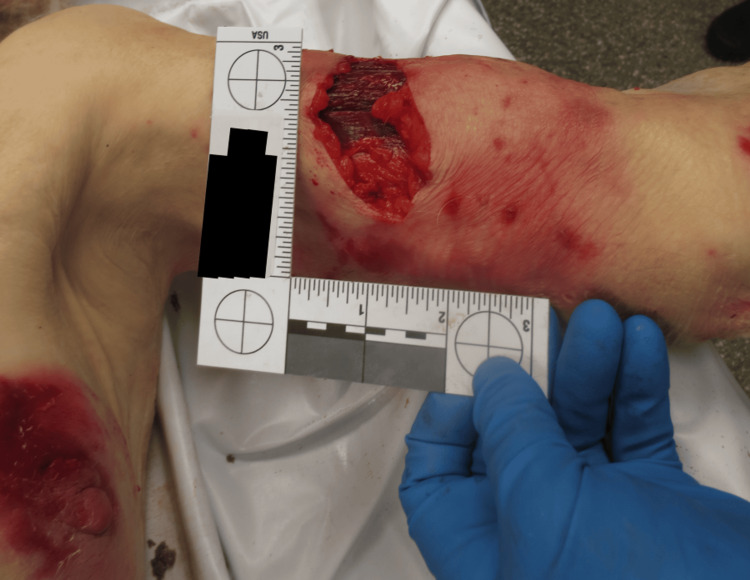
Avulsion/laceration injury to the left arm with surrounding puncture wounds.

Autopsy findings revealed a 10 x 7 x 5 inch pulverizing injury to the medial right thigh, penetrating into musculature and transecting the femoral artery and vein, as seen in Figures 1-2. Additional canine-induced trauma included abraded lacerations on the limbs, torso, and face, with generalized contusions. Internal examination revealed mild frontotemporal cerebral atrophy consistent with dementia. Toxicology was measured as positive for donepezil and escitalopram at therapeutic doses. The cause of death was exsanguination as a result of injury inflicted by the canines, and the manner of death was designated as accidental.

Case 2

A 68-year-old female who lived in Costilla County was found deceased on January 23, 2025. She had a previous medical history significant for cerebrovascular disease, traumatic brain injury, lung cancer, and cardiovascular disease. She was found half-naked, her garments were shredded, and a scuffle was obvious. Investigators from Colorado Parks and Wildlife and the Colorado Bureau of Investigation were involved due to the possibility of a wild or feral animal attack.

**Figure 3:**
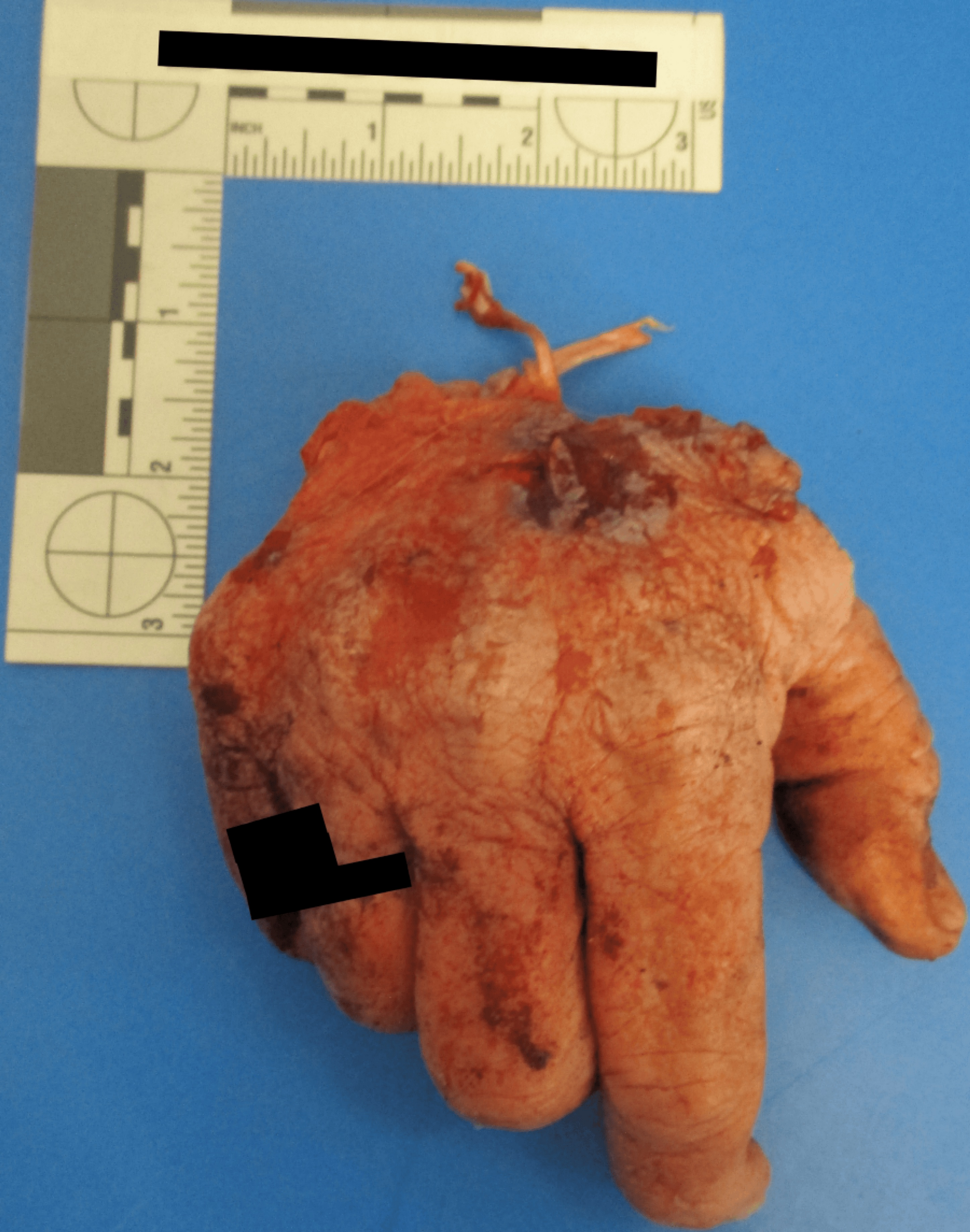
Avulsed right hand with vascular pedicle stump visible at the wrist.

**Figure 4:**
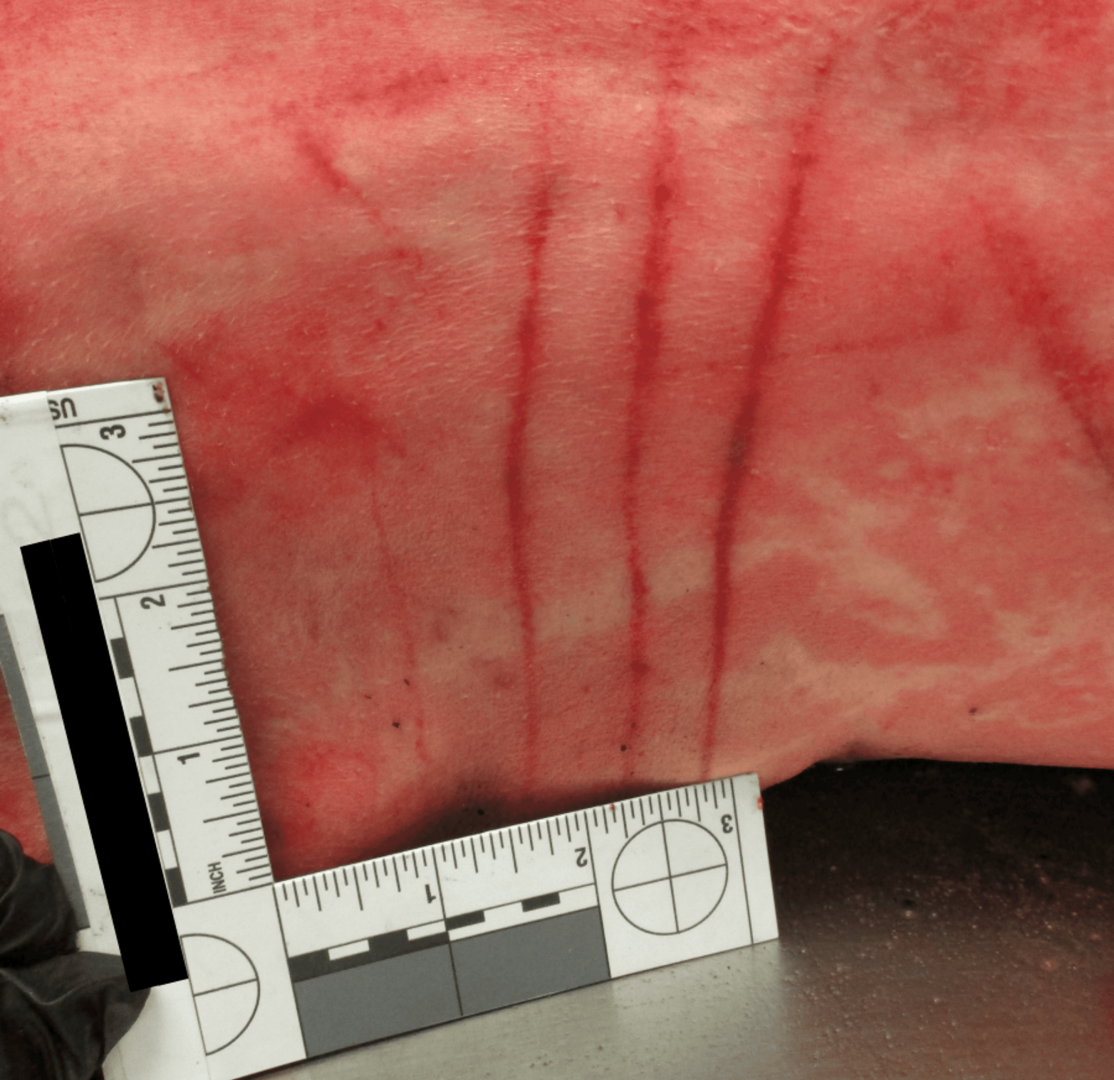
Parallel linear abrasions suggestive of claw or tooth dragging.

Autopsy revealed extensive bite and scratch injuries across the face, torso, arms, and legs, with numerous puncture wounds and vital reaction. Notably, the right hand was avulsed (Figures 3-4), and both upper limbs showed significant destruction. The injuries were consistent with a sustained and violent mauling. Forensic analysis noted paired puncture wounds, irregular avulsions, and circumferential limb trauma typical of canine dentition and tearing behavior. The absence of claw marks or a singular neck-focused bite excluded large felids or other wild predators as the source. Internal examination confirmed chronic left parietal stroke and lung carcinoma, but no acute internal cause of death. The cause of death was determined to be due to multiple injuries from an animal attack, consistent with canine-induced trauma. The manner of death was ruled an accident. 

Case 3

A 57-year-old male who lived in Conejos County was found deceased on February 19, 2025. The deceased was walking his dog preceding the attack, which was his daily routine. Neighborhood witnesses reported that the man and his dog were attacked by a neighbor’s pitbulls after a dog-on-dog confrontation. The decedent was found unresponsive after the attack with significant injuries to the head and extremities. His dog was also reportedly killed during the incident.

**Figure 5:**
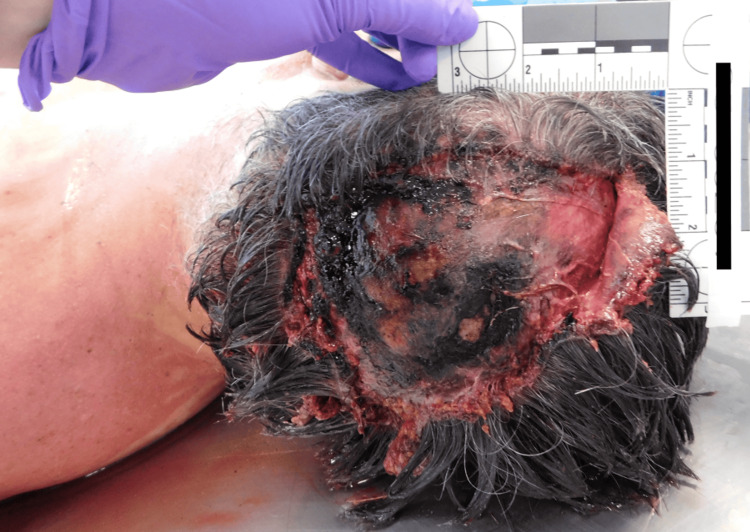
Avulsion injury to the occipital scalp with deep tissue destruction.

Autopsy documented widespread abrasions, puncture wounds, and incised injuries to the scalp, face, arms, and legs. A large section of the occipital scalp was avulsed, with deep soft tissue gouging and evidence of tendon exposure (Figure 5). Defensive wounds were noted on the forearms and hands, suggesting attempts to intervene or protect his dog. Internal examination revealed no significant comorbidities or toxicologic contributors. The cause of death was concluded to be dog mauling, with the manner of death certified as an accident. Table 1 presents a summary of victim and scene characteristics in the three fatal dog mauling cases.

**Table 1:** Summary of victim and scene characteristics in the three fatal dog mauling cases. CA, Cancer; TBI, Traumatic Brain Injury

Case	Age/Gender	Comorbidities	# and Type of Dogs	Location	Key Injuries	Mode/Manner of Death
1	76/F	Dementia	14 small-medium breeds	Home	Femoral artery laceration, soft tissue trauma	Exsanguination, accidental
2	68/F	Stroke, lung CA, TBI	Suspected feral dogs	Outdoors	Right-hand avulsion, extensive upper limb damage	Animal attack, accidental
3	57/M	None significant	Two pit bulls	Neighborhood	Scalp avulsion, limb gouging	Dog mauling, accidental

## Discussion

Comparative analysis

The three cases presented in this series highlight both shared forensic features and distinct contextual differences in fatal dog maulings. A comparative review of injury distribution, scene dynamics, victim characteristics, and contributing factors reveals important patterns relevant to forensic assessment and public health interventions.

Injury patterns and mechanisms

In all three cases, the decedents had received classic signs of canine-inflicted trauma: puncture wounds, abrasions, and lacerations characteristic of repeated biting, tearing, and pulling. A significant finding was that each decedent exhibited significant soft tissue damage to the extremities and head/neck region. Case 1 had a gross lethal injury, a crushing wound to the right medial thigh that traversed the femoral vein and femoral artery and resulted in exsanguination. Case 2 comprised full avulsion of the right hand and extensive injury to both upper extremities, and Case 3 comprised avulsion to the back of the head, facial injury, and limb injury with tendon exposure. These wounds are characteristic of both predatory and defense-related bite activity.

Scene context and canine involvement

The circumstances surrounding each fatality differed significantly. Case 1 occurred within the victim’s own home, with 14 familiar dogs involved. Though no specific trigger of the event was identified, the attack may have been provoked by confusion, accidental provocation of the animals, or a fall. Case 2 occurred in a remote outdoor location, with the attacking animals presumed to be feral dogs. The nature of the attack prompted the involvement of state investigators and wildlife officials. Case 3 occurred during a routine dog walk in which a neighbor’s pit bulls attacked, likely due to a dog-on-dog provocation.

This variation in case presentation exemplifies the importance of rigorous scene investigation and canine behavior profiling. The number of dogs involved also varied: Case 1 involved a large pack of small-to-medium domestic breeds, whereas Case 3 involved two large dogs of a historically aggressive breed. While Case 2’s attackers were not directly identified, the severity and distribution of injuries support a sustained multi-canine assault.

Victim factors and contributing conditions

Each case included a victim over the age of 55 with varying degrees of physical vulnerability to the attacks. Case 1 included a past medical history of advanced age and dementia, which likely contributed to her inability to defend herself or escape the attack. Case 2 involved multiple comorbidities, such as stroke, lung cancer, and prior brain injury, which may have affected defense capabilities towards the attack. Case 3 was comparatively younger and healthier but faced multiple dogs and sustained extensive injuries before discovery.

Toxicology reports were largely unremarkable across all cases, with only one case (Case 1) testing positive for therapeutic doses of prescribed medications (donepezil and escitalopram). None of the cases showed involvement of alcohol or illicit drugs. 

Forensic and medico-legal implications

All three cases were certified as accidental deaths, despite the presence of aggressive behavior by owned dogs in two cases. These determinations reflect not only the pathology but also the investigative context, particularly owner responsibility, containment of animals, and whether negligence or provocation played a role. From a forensic perspective, this series highlights the importance of recognizing bite wound morphology, assessing the distribution and severity of injuries in the context of defense or predation, considering victim vulnerability and behavioral incapacity, and collaborating with animal control or wildlife agencies when canines are unidentified or stray.

Forensic interpretation of canine injuries

All three cases demonstrated classical features of canine-inflicted trauma: puncture wounds, soft tissue avulsion, and irregular lacerations consistent with biting and tearing behavior. These findings align with prior descriptions of dog bite fatalities, particularly when patterned injuries and tissue gouging are present [9,10]. Recognition and interpretation of such patterned wounds remain a cornerstone of forensic assessment, as correlating morphology with causative mechanisms strengthens medico-legal determinations. Comparable insights have been reported in sharp-force contexts, such as injuries produced by a modified sickle, where unique patterned imprints aided weapon identification and highlighted the broader value of linking wound morphology with causative mechanisms [11]. However, distinguishing ante- from postmortem injuries can be particularly challenging in the absence of witnesses or video evidence, especially when scavenging behavior has occurred [4]. Scene investigation and forensic imaging are thus essential complements to autopsy in attributing injuries to animal rather than human origin [6].

Vulnerable populations and environmental risk factors

All victims in this series were elderly individuals, two with pre-existing medical conditions that may have contributed to reduced escape or defense capacity. This aligns with epidemiological patterns identifying children and older adults as high-risk groups for fatal canine encounters, due to impaired mobility and delayed recognition of threats [1,2]. Elderly individuals living alone with multiple dogs, particularly when cognitively impaired, represent a population of emerging concern in both forensic and public health contexts [12].

Multi-dog dynamics and scene complexity

Two of the cases involved multiple dogs, with one attack implicating more than a dozen animals. Prior literature suggests that pack behavior among domestic dogs can amplify aggression, leading to more extensive and prolonged injuries [6,10]. Group attacks may also delay intervention, particularly in rural settings or when victims are unable to call for help. These dynamics raise broader regulatory questions about the oversight of multi-dog households, especially in contexts where owners are unable to maintain control [13].

Breed and ownership implications

The involvement of pit bull-type dogs in one of the cases continues to reflect their disproportionate appearance in national fatality statistics [1,3]. Nonetheless, one fatality in this series involved smaller mixed-breed dogs, reinforcing the principle that breed alone is not predictive of fatality risk [14]. As supported by a systematic review of bite-related deaths, behavioral history, context, and the owner’s ability to control the dog are more critical variables than breed alone [4]. This further underscores the need for individualized forensic assessments rather than reliance on breed-specific assumptions.

Forensic best practices

These cases support several best practices in the forensic evaluation of suspected dog maulings. A comprehensive external examination of patterned wounds is essential, especially those with vital reaction. Careful documentation of defensive injuries is also important, as these may suggest the decedent’s level of awareness or struggle. Coordination with animal control, law enforcement, and wildlife agencies is recommended to correlate bite marks with potential suspected attackers. Additionally, consideration of underlying conditions and toxicology is necessary to assess the victim’s capacity to respond. Though none of these cases held alcohol or drug use as significant to the case, their inclusion in the toxicology analysis remains crucial. The involvement of such substances can help with the understanding of potential impairment, especially when the scene circumstances are ambiguous.

Standardization of terminology

During the review of these three cases, it became evident that inconsistent terminology was used across forensic documents. For example, three phrases were utilized for the attacks, including “canine-induced trauma,” “dog mauling,” and “dog bite fatality” by three separate physicians. Discrepancies in terminology usage may hinder the ability to aggregate data, track trends, and affect public health reporting [15]. Several improvements in forensic pathology reporting can be gained from standardizing terminology. Such improvements include enhancing the clarity of forensic reports, improving interagency communication, and supporting future research in this field. We advocate for a consistent use of the term “canine-induced trauma” to encompass the range of dog attacks, including single and multi-dog attacks. 

Public health and policy implications

These cases, though ruled accidental, raise broader concerns in the public health and regulatory fields. Currently, Colorado enforces “dangerous dog” statutes across the state to prevent the ownership of canines with histories of attacks that inflict damage. However, leash laws and limitations on the number of dogs permitted per household vary across municipalities, creating an inconsistent legal environment for canine control. Additionally, it is likely that actual enforcement and public awareness of these laws vary widely by county. These findings may highlight the necessity to increase oversight of multi-canine households and unrestrained dogs, as well as increase owner education regarding vulnerable populations (e.g., the elderly or cognitively impaired). Stronger reporting protocols and increased community-based education may help prevent future similar fatalities.

## Conclusions

This comparative review of three fatal dog maulings in Colorado illustrates both the wide variation in scene circumstances and the consistent severity of injuries caused by dogs. Across settings ranging from household pets to feral or unrestrained animals, all victims suffered devastating soft tissue trauma, including avulsion and patterned puncture wounds, with defensive injuries noted in two cases. The elderly and those with significant medical conditions appeared particularly vulnerable.

These findings highlight the importance of collaboration between forensic pathologists, public health officials, animal control, and policymakers to improve the prevention and investigation of fatal dog attacks. Greater consistency in forensic terminology, more focused public education, and stronger animal control measures may help reduce risk and strengthen community response in high-risk populations.
